# Using Intervention Mapping to Develop a Decision Support System–Based Smartphone App (selfBACK) to Support Self-management of Nonspecific Low Back Pain: Development and Usability Study

**DOI:** 10.2196/26555

**Published:** 2022-01-24

**Authors:** Malene Jagd Svendsen, Louise Fleng Sandal, Per Kjær, Barbara I Nicholl, Kay Cooper, Frances Mair, Jan Hartvigsen, Mette Jensen Stochkendahl, Karen Søgaard, Paul Jarle Mork, Charlotte Rasmussen

**Affiliations:** 1 The National Research Centre for the Working Environment Copenhagen Denmark; 2 Department of Sports Science and Clinical Biomechanics University of Southern Denmark Odense Denmark; 3 Health Sciences Research Centre UCL University College Odense Denmark; 4 Institute of Health and Wellbeing General Practice & Primary Care University of Glasgow Glasgow United Kingdom; 5 School of Health Sciences Robert Gordon University Aberdeen United Kingdom; 6 Chiropractic Knowledge Hub Odense Denmark; 7 Department of Clinical Research University of Southern Denmark Odense Denmark; 8 Department of Public Health and Nursing Norwegian University of Science and Technology Trondheim Norway

**Keywords:** intervention mapping, behavior change, low back pain, self-management, mHealth, app-based intervention, decision support system, digital health intervention, mobile phone

## Abstract

**Background:**

International guidelines consistently endorse the promotion of self-management for people with low back pain (LBP); however, implementation of these guidelines remains a challenge. Digital health interventions, such as those that can be provided by smartphone apps, have been proposed as a promising mode of supporting self-management in people with chronic conditions, including LBP. However, the evidence base for digital health interventions to support self-management of LBP is weak, and detailed descriptions and documentation of the interventions are lacking. Structured intervention mapping (IM) constitutes a 6-step process that can be used to guide the development of complex interventions.

**Objective:**

The aim of this paper is to describe the IM process for designing and creating an app-based intervention designed to support self-management of nonspecific LBP to reduce pain-related disability.

**Methods:**

The first 5 steps of the IM process were systematically applied. The core processes included literature reviews, brainstorming and group discussions, and the inclusion of stakeholders and representatives from the target population. Over a period of >2 years, the intervention content and the technical features of delivery were created, tested, and revised through user tests, feasibility studies, and a pilot study.

**Results:**

A behavioral outcome was identified as a proxy for reaching the overall program goal, that is, increased use of evidence-based self-management strategies. Physical exercises, education, and physical activity were the main components of the self-management intervention and were designed and produced to be delivered via a smartphone app. All intervention content was theoretically underpinned by the behavior change theory and the normalization process theory.

**Conclusions:**

We describe a detailed example of the application of the IM approach for the development of a theory-driven, complex, and digital intervention designed to support self-management of LBP. This description provides transparency in the developmental process of the intervention and can be a possible blueprint for designing and creating future digital health interventions for self-management.

## Introduction

Low back pain (LBP) is well-documented as one of the most common reasons for activity limitation, sick leave, and disability [1-4]. Clinical guidelines for LBP consistently endorse patient education, general physical activity, exercise, and the promotion of core self-management components of frontline care [5-9]. However, implementation of self-management may be challenging, perhaps because of its multifaceted complex nature, with several interacting components and health care settings. Therefore, new effective ways of delivering supported self-management for people with LBP are needed.

Digital health interventions (DHIs), such as those that can be provided by smartphone apps, have been proposed as a promising mode for supporting self-management in people with chronic conditions [10]. In a recent systematic review on the use of DHIs for supporting self-management of LBP, we found that the literature was heterogeneous in terms of reporting intervention details, making it difficult to understand what might work best, for whom, and in what circumstances [11]. The descriptions of the intervention development and use of theory were either brief or completely lacking in all the included studies, and the evidence base for DHIs to support self-management of LBP was weak [11]. In another systematic review, Garg et al [12] found that web-based interventions for supporting individuals with LBP were useful. In particular, interventions that offer feedback or tailoring based on user responses and elements from cognitive behavioral therapy seem to be beneficial; however, the effectiveness of DHIs in supporting self-management of LBP remains unclear [12]. A systematic review of smartphone apps for self-management of LBP concluded that researchers, health care professionals (HCPs), people with LBP, and app developers need to work closely together to develop DHIs that are accurate, evidence-based, and engaging [13]. Structured intervention mapping (IM) provides a well-defined framework for the development, implementation, and evaluation of interventions by integrating the target population and stakeholders in the process [14]. Using IM for development also fits with the Medical Research Council framework for evaluation of complex interventions [15] and CONSORT-EHEALTH (Consolidated Standards of Reporting Trials of Electronic and Mobile Health Applications and Online Telehealth) [16] guidelines for reporting DHIs. One of the issues emphasized by CONSORT-EHEALTH is that a detailed description and documentation of the intervention is required to fully understand its effectiveness [16]. The stepwise process of IM provides just that, and it has previously been used to develop and adapt evidence-based self-management programs in other settings [17,18]. selfBACK [19], a case-based reasoning (CBR) DHI, which is delivered as an app, is designed to improve the self-management of nonspecific LBP to reduce pain-related disability. The selfBACK app provides weekly tailored self-management plans (SMPs) targeting physical activity, strength and flexibility exercises, and education. In addition, the app also provides access to a variety of tools and information on the management of LBP that the participants can use at their convenience. A randomized controlled trial (RCT) is examining the effectiveness of the intervention as an add-on to usual care [20]. The aim of this paper is to describe the IM process used to design and create selfBACK.

## Methods

### IM Approach

IM is a 6-step process for the development, implementation, and evaluation of an intervention; however, here we have described just 5 steps as step 6, the planning of the evaluation of the intervention, is published as a protocol for an RCT [20] alongside an implementation and process evaluation protocol [21]. Each step comprises several tasks, which, once completed, inform the next step, as detailed by Bartholomew et al [14]. The IM approach considers the target population, its surrounding environment, and the persons in that environment who might influence the target population. It aims to facilitate participation and consultation of stakeholders and provides a structure for the integration of theory, findings from empirical literature, and information collected from the target population. Throughout all the steps of the development phase, we used *core processes* (ie, posing questions, brainstorming, reviewing empirical literature, reviewing theories, assessing needs for new data, and developing a working list of answers) as the underlying methods [14].

### Step 1: Logic Model of the Problem

First, we established a planning group comprising partners from both clinical and research backgrounds and those with expertise in app development and health innovation management. The planning group comprised physiotherapists, chiropractors, physicians, exercise physiologists, behavioral scientists, computer scientists, and app designers.

We conducted a needs assessment from a societal and user perspective as a response to a European Union Horizon 2020 program calling for “self-management of health and disease and decision support systems based on predictive computer modelling used by the person him or herself.” To meet the criteria in the call, the project aimed solely for an intervention targeting individuals with LBP and not their surrounding environment or the persons in that environment. The needs assessment was informed by 2 systematic literature reviews [11,22] that we conducted on digital interventions for supporting self-management of LBP, reviews of clinical practice guidelines, extensive supplementary literature searches on factors associated with LBP and self-management, interviews with patients with LBP and HCPs, clinical experience, and group discussions in the planning group.

The process resulted in a logic model of the problem that mapped personal determining factors of LBP self-management, adverse behavior in relation to self-management and how that behavior affects LBP and associated health issues, and, finally, the impact on quality of life. Subsequently, based on recommendations from the LBP literature and previous experiences within the planning group with interventions targeting LBP, the overall program goal of the intervention was formulated.

### Step 2: Program Outcomes and Objectives—Logic Model of Change

The program outcomes were formulated based on the scrutiny of systematic reviews, overview papers, expert opinion papers on core outcome domains, and outcome measures used in previous LBP interventions. To identify behavior-related outcomes, we consulted the literature on behavior change theory related to pain, pain-related disability, and self-management. We also used information from the 2 systematic reviews that we conducted on digital interventions to support self-management of LBP [11,22]. Building on the results from step 1 and an examination of the literature, a logic model of change specifying the outcomes and objectives was developed. The model included a definition of the change in behavior needed for improvement in LBP (*behavioral outcomes*) and detailed specifications of what people with LBP need to do to perform that behavior (*performance objectives*). Finally, a matrix pairing the performance objectives with the determining factors of LBP self-management identified in step 1, with positive statements about what needs to occur to achieve the performance objectives (*change objectives* [14]), was created.

### Step 3: Program Design

In step 3, the intervention concepts were outlined in an iterative process of matching relevant behavioral change theory to the practical application ideas. Clinical practice guidelines and their accompanying patient leaflets, as well as pain management websites deemed to originate from trustworthy sources, were reviewed to outline the intervention content. The choice of specific physical exercises and educational content was determined in a parallel process with the GLA:D Back project, a group education and exercise program that translates guideline recommendations into a clinician-delivered package for the promotion of self-management in people with persistent or recurrent back pain [23]. A psychologist provided feedback on the educational content. The process of identifying theories of behavior change that would guide the choice of methods and practical applications of these methods was informed by the review of behavior change theory from step 2 and the extensive experience with trials on self-management of LBP among the planning group. Parts of the interviews conducted for step 1 provided valuable information on users’ needs and wishes regarding the self-management app. We also purposefully reviewed the literature on practical ideas for enhancing engagement with the app, for example, as gamification and notification systems. For all determinants and performance objectives, theoretically underpinned behavior change techniques were chosen, and the practical application of each technique was brainstormed, discussed, and refined by the planning group. The outcome of this third step was an outline of the intervention themes, components, and sequences of the intervention.

### Step 4: Program Production

All technical and practical features of delivering the selfBACK intervention through a smartphone app and the participant documents for use in the RCT study were created in step 4 [24,25]. Throughout the production process, brainstorming sessions and workshops among planning group members for production of content were held continuously, and technical solutions for running the digital intervention (ie, tailored decision support for self-management using artificial intelligence) were developed, tested, and refined. Furthermore, the user interface was designed, starting with wireframes, visual identity and design, and functional requirements. Before we designed the app’s visual identity, we performed a search of existing apps on Google Play and the Apple App Store. The apps were qualitatively evaluated using Apple Human Interface Guidelines, Apple Research Kit, and Google’s Material Design. These guidelines are considered state-of-the-art documents and trendsetters in app design. As part of the design process, we created fictional characters (personas) based on the literature scrutiny from the previous steps and the interviews conducted in step 2. The personas helped the app developers understand the needs, experiences, behaviors, and goals of patients with LBP and guide the design process. The user journey map was matched with the personas. This map served as a blueprint for the design and development phases of the app. The map depicted an overview of the journey that a user would embark on from experiencing LBP for the first time to finding an individual solution to their pain and how that journey may be guided toward using the selfBACK app.

A total of 2 feasibility studies with target population participants were conducted: 1 in the United Kingdom and 1 in Norway. The UK feasibility study (n=16) was conducted to explore the feasibility and acceptability of the baseline questionnaire, physical activity monitoring, and feedback strategies with a prototype app, whereas the Norwegian feasibility study (n=10) tested the full intervention with an early version of the selfBACK app [26]. In both the UK and Norwegian feasibility studies, we applied quantitative and qualitative methods to inform further development of the intervention. Simultaneously, app prototypes were tested by the planning group members and external users conducted both as group sessions and as real-time tests of app use in consecutive periods (n=65 female, 60 male). Finally, a pilot study in Denmark and Norway (n=51) was conducted using a complete version of the app to test recruitment and screening procedures and inform the design of the effect and process evaluation [27]. Intervention content was developed and continually refined in an iterative process over a period of >2 years. To ensure consistency throughout the app content and the trial documents, everything was first completed in English and then translated to Danish and Norwegian (settings for the RCT study).

### Step 5: Implementation Plan

The plan for the implementation and adoption of the selfBACK app was based on the results from previous steps, the pilot study, and theory, taking into account both behavior change and implementation theories, to improve the likelihood of embedding the DHI into daily routines. The program use outcomes (adoption and implementation) were specified. The selfBACK app targets care-seeking patients; therefore, it was necessary to also consider the adoption of the selfBACK app from the recruiting HCPs’ perspective. We used normalization process theory (NPT) to identify determinants of adoption and implementation and linked them to each performance objective. Finally, a matrix was created by planning the group discussions. We used behavior change techniques (BCTs) to convert the change objectives into practical strategies. Recruitment material for patients with LBP (app users) and recruiting clinicians was produced together with a plan of how to attract both groups to the project. Procedures for the recruitment of and initial and sustained contact with the HCPs were established, as well as the procedures for inclusion, screening, randomization, follow-up, and evaluation of the app users [20].

## Results

### Step 1: Logic Model of the Problem

In step 1, we interviewed 8 patients with LBP (5 [63%] men and 3 [27%] women) about their experience with treatment of LBP and how they usually self-managed their LBP. From these interviews, we identified and prioritized *not following evidence-based self-management strategies* as the most important changeable, adverse behavioral factor contributing to poor outcomes for people with LBP. This behavior is believed to be affected by the following personal determining factors: not being aware of ways of self-managing LBP (*lack of knowledge and awareness*); not possessing skills or being insecure about the ability to self-manage LBP (*low self-efficacy*); or fearing an increase in pain when doing physical activities (*fear-avoidance behavior and catastrophizing*), negative expectations about the course of LBP (*low outcome expectations*), and challenges encountered when trying to fit self-management strategies within the context of daily life (*low motivation*). In addition, the 2 interviewed HCPs reported that many patients relied on HCPs to cure their pain and that getting people to change their perception of how best to manage LBP (eg, avoid bed rest and stay active) was one of the biggest problems for HCPs (patients’ *lack of knowledge* and *low self-efficacy*). Convincing people that simple at-home body weight exercises and not gym memberships could be helpful was also challenging (*low motivation*). All of this informed a logic model of the problem from the target population’s perspective. Subsequently, the planning group decided that the overall program goal was *to improve the self-management of nonspecific LBP to reduce pain-related disability*. The personal determinants were knowledge and awareness, skills, fear avoidance and catastrophizing, self-efficacy, and motivation and outcome expectations.

### Step 2: Program Outcomes and Objectives—Logic Model of Change

In this step, we created a matrix specifying the intended behavioral change expected to result from selfBACK intervention. The result was a schematic representation of what patients with LBP needed to do to reach the overall objective of self-managing their LBP, as exemplified in Table 1.

**Table 1:** Extract of the matrix of change objectives for the following behavioral outcome: To increase use of evidence-based self-management strategies. The full matrix is available in Multimedia Appendix 1 (Table S1).

Behavioral outcome^a^		Personal determinants				
		Knowledge and awareness^b^	Skills^c^	Fear avoidance and catastrophizing^d^	Self-efficacy^e^	Motivation and outcome expectations^f^
**PO^g^** **1: Accept self-management as treatment strategy for LBP^h^** **and make the decision to self-manage LBP with support from selfBACK app**						
	**Change objectives**	Identify positive characteristics of self-management and negative characteristics of provider dependent behavior List examples of self-management of LBP	Demonstrate ability to operate selfBACK app	Recognize fearful thoughts and negative thinking in relation to self-management	Express confidence in the ability to operate selfBACK app	Express positive feelings or thoughts about engaging in self-management of LBP Expect that self-managing will ease living with LBP and achieving life goals
**PO^g^** **14: Integrate self-management strategies for LBP^h^** **into daily life**						
	**Change objectives**	List ways to integrate self-management of LBP into daily routines	Demonstrate ability to schedule self-management into daily routines	Recognize fearful and negative thoughts and feelings in relation to integrating self-management into daily routines Recognize own fear-avoidance behavior in relation to integrating self-management into daily routines	Express confidence in the ability to integrate self-management of LBP into daily routines	Express positive feelings or thoughts about integrating self-management into daily routines Expect that integration of LBP self-management will lead to a healthier, better life

^a^Increase use of evidence-based self-management strategies.

^b^Increase knowledge of self-management behavior.

^c^Develop ability to engage in self-management behavior.

^d^Reduce fear or negative expectancies about engaging in self-management behavior.

^e^Improve perceived ability to uptake and engage in self-management behavior.

^f^Improve autonomous motivation to engage in self-management behavior and improve expectations to the outcome of self-management behavior.

^g^PO: performance objective.

^h^LBP: low back pain.

### Step 3: Program Design

#### Core Components

Using evidence for the 3 main intervention components from clinical guidelines [5-8,28-31] and studies on management of LBP [32], as well as patient leaflets from the National Institute for Health and Care Excellence [33-41] and LBP management websites [42-45], three overall themes emerged: physical exercise, education, and physical activity.

##### Physical Exercise

Guidelines de campo [28,46] and systematic reviews on LBP treatment [47-53] endorse physical exercises for the management of persistent LBP. There is no evidence that any one type of exercise is better than others; however, strength training and motor control exercises are most commonly used [54,55]. *Exercises for flexibility* that aim to restore or improve the range of motion of the lumbar spine are also often part of programs to alleviate LBP [47,48,53]. In addition, reviews on motor control exercise support another type of exercise, *pain relief,* as being effective to support management of pain [47,50,52,53,56]. These exercises comprised movements performed in the midrange and without strong muscle contractions to facilitate controlled and smooth movements.

The amount of exercise was not always clearly described in RCTs; however, longer durations of exercise periods and heavier training seemed to be more effective in reducing back pain compared with shorter exercise periods and lighter loads [57]. The American College of Sports Medicine recommends 2 to 3 weekly sessions for muscle training at 60%-70% of 1 repetition maximum for beginners and 80% or 1 repetition maximum, sets of 8 to 12 repetitions for strength and power, and >15 for endurance for those who are more experienced [58]. To maintain a good range of motion, flexibility exercises to the end range are recommended 2 to 3 days a week, held for 30 seconds, and repeated 2 to 4 times [58]. As the literature does not clearly indicate the most effective exercises, we chose to design individually tailored exercise programs based on, for example, symptoms, preferences or fitness levels, and exercises aimed at pain control or pain reduction, improved motor control, strength, and flexibility. Consequently, exercises included in the selfBACK exercise bank were categorized into six different targets: (1) flexibility exercises, (2) pain-relieving exercises in addition to strength exercises, (3) back extensors, (4) gluteal and hip muscles, (5) abdominal muscles, and (6) core muscles. The organization of exercises by their target, rules for progression, or regression between exercise levels were guided by consensus discussions among experienced clinicians and researchers, physiological reasoning, and clinical experience.

##### Education

A cognitive behavioral approach to patient education revolving around teaching or promoting pain coping skills such as activity pacing and progression guidance, goal setting and action planning, and mindfulness techniques [30], as well as reassurance about the prognosis of LBP [31], was recommended by the guidelines [28,29,31]. Furthermore, we chose education that comprised information about the condition, consequences and management, discouragement of bed rest, and advice to stay physically active. Our systematic reviews, conducted in step 1, also highlighted the importance of providing support that was easy for a user to integrate within the many competing activities of their daily lives [11,22]. Themes in relation to education, which were extracted from the guidelines [5-8,28-30], patient leaflets [33-41], and pain management websites [42-44], were organized under the main and subthemes, as presented in Textbox 1.

**Main themes and subthemes of the educational content.**

**First aid for acute back pain**

- First aid reassurance
- First aid stay active

**Fitting self-management into your daily life**

- Daily activities
- Me time

**General information about low back pain**

- Cause of low back pain
- Guidelines low back pain
- Imaging
- Pain rating
- Reassurance
- Start exercise
- Stay active
- Structure of back

**How body and mind are connected**

- Mind–body connection

**How thoughts, behavior, attitudes, and feelings affect low back pain**

- Accepting pain
- Anxious thoughts and feelings
- Attitude
- Changing negative thoughts
- Distraction
- Distress
- Fear avoidance
- Stress
- Thoughts

**How to overcome barriers for self-management of low back pain**

- Barrier facilities
- Barrier family and work
- Barrier time
- Barrier tiredness
- Barrier support
- Barrier weather

**How to practice mindfulness for low back pain**

- Mindfulness

**How to reach your goals**

- Pacing

**How to seek social support**

- Family and friends
- Work

**How to set specific, measurable, achievable, realistic, and timely (SMART) goals**

- Action planning
- Goal setting

**How to sleep better at night**

- Sleep tips

**How to solve problems**

- Problem solving

**Low back pain and other medical conditions**

- Anxiety
- Depression
- Musculoskeletal pain
- Sleep problems

**Self-manage your low back pain**

- Encouragement to self-management

##### Physical Activity

The importance of physical activity has been recognized as a prime strategy in guidelines for self-management of LBP, including advice to stay active and at work, as well as discouragement of bed rest [5-8,28-30]. This has, to a large part, been based on the deconditioning model of LBP [59]; that is, patients with LBP may be restricted in the performance of everyday physical activities. Consequently, they risk developing an inactive lifestyle, and a vicious circle may then gradually develop. The recommended daily step count was based on the general recommendations for physical activity [58]. A minimum step count goal of 3000 per day was chosen to reflect the fact that participants may have functional disabilities that affect their physical activity level. Optimally, users should reach 10,000 steps per day [58,60].

#### Outline of Intervention App Design

**Figure 1:**
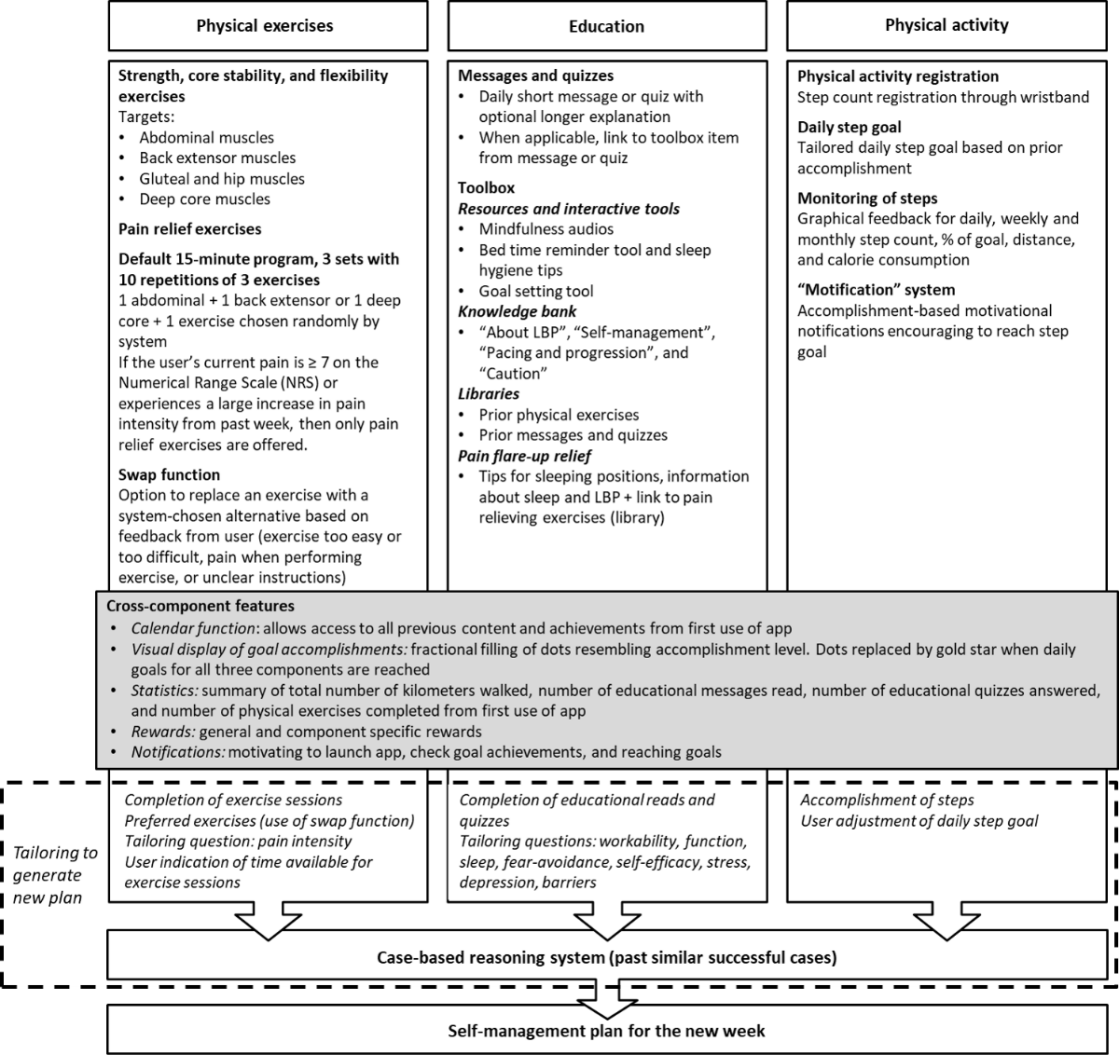
Outline of the selfBACK intervention. Tailoring questions presented in Sandal et al [20]. LBP: low back pain.

The blueprint of the selfBACK intervention content that is incorporated in the selfBACK app is shown in Figure 1. Weekly SMPs with content from the three main intervention components are outlined as follows: (1) a bank of physical exercises; (2) a bank of educational messages and quizzes; and (3) physical activity registration in terms of step counts, together with an accomplishment-based motivational notification system. The bank of physical exercises has 59 strength and flexibility exercises organized in 5 targets with up to 6 difficulty levels and 11 additional pain relief exercises. Instructions for each exercise were provided in text and video formats, with real-life models demonstrating the exercises (no audio). Exercise sessions were recommended to be performed 3 to 5 times a week. The default program comprised 3 exercises constituting 15 minutes per session. An exercise could be swapped with another chosen by the system based on the participants’ reason for swapping (eg, exercise too difficult or easy, pain when performing, or unclear instructions). The educational material comprised daily messages or quizzes and a toolbox. Messages and quizzes were structured under the 14 main themes with up to 9 subthemes identified in step 2 (Textbox 1). Short messages (<140 characters) were followed by an optional long message (maximum 500 characters), which included a more thorough explanation. Quizzes with yes or no answers were followed by the correct answer and an explanation. When applicable, the messages were accompanied with a link to a toolbox item with additional content to support self-management, that is, interactive tools (goal setting or bedtime reminder), mindfulness audios, and an explanatory text about how LBP, self-management, and the selfBACK app were connected. Additional toolbox items included 2 libraries with all previous physical exercises and educational messages or quizzes and advice on handling LBP relapse. Physical activity (step count) was tracked using a wearable device (Mi Band 3, Xiaomi Corp) connected to the app via Bluetooth. Daily, weekly, and monthly accomplishment of steps were graphically available to the participant. On the basis of their accomplishments, motivational notifications (*motifications*) encouraged participants to reach their daily step count goal.

Furthermore, the app had five cross-component features:

- A calendar function allowed users to access content and see accomplishments from the first day of using the app.
- Dots that gradually filled up as the daily goals were reached displayed the accomplishment level. On days where all 3 goals were reached, a gold star replaced the dots.
- A statistical summary showed the distance walked and number of messages read, quizzes answered, and exercises performed since the start of using the app.
- General and component-specific rewards were unlocked once the users performed the goal. For example, reaching all daily goals for the first time gave a reward. The next levels were 3, 7, and 14 times; another example was the number of steps in total, where the first reward was at 50,000 steps, followed by 100,000, 200,000, and 800,000 steps.
- Notifications motivated users to engage with the app but could be turned off by the user. Examples of these *motifications* were “Fantastic performance today! Achieved your step count goal!” and “You’re more than half way to your step-count goal. Taking the stairs instead of the lift can really help towards your step count goal.”

Tailoring of SMPs to individual participants was based on data from four sources: (1) the baseline questionnaire [20]; (2) the participant’s achievement of physical exercises in the preceding week; (3) the participant’s achievement of steps in the preceding week; and (4) weekly tailoring of question and answer sessions with a changing selection of questions from the baseline questionnaire [20], the participant’s indication of time available for physical exercise per session, the participant’s preference for exercises, and the participant’s adjustment of the step goal for the new week (–10% to +10% to 20% from what the app suggested based on the preceding week), with an upper limit of 10,000 steps. A CBR system supported by a sophisticated rule engine used the abovementioned data from the current participant (case) and previous similar participants (cases) to generate new SMPs [19,25]. Generation of a new SMP was initiated by the weekly tailoring session or upon return to the app if the participant had not used the app for more than a week. This process is described in detail elsewhere [19,25].

#### Underpinning Theories for Behavior Change and Engagement in DHIs

Self-management interventions can be characterized as behavior change interventions in that they are designed to help the patient learn and adopt a set of health behaviors and thus, benefit their condition [61]. To underpin the selfBACK intervention theoretically in relation to behavior change and engagement, 2 frameworks were applied in the design. The Transtheoretical Domain Framework (TDF) is an overall framework encompassing 33 behavior change theories [62]. TDF has been validated for use in behavior change and implementation research [63]. To facilitate the application of BCTs [64,65], matrices have been created by expert consensus, mapping BCTs and theoretical constructs as per TDF [66,67]. Furthermore, NPT underpins the strategies for uptake and adherence to the intervention. NPT is a sociological theory that has been widely used to understand the factors that influence how technologies or therapies are implemented, embedded, and integrated into daily routines [68-70]. NPT has four main constructs: (1) coherence—the sense-making work that participants undertake, which influences whether they are willing to embed a new practice in their lives; (2) cognitive participation—the work that participants undertake to engage with the new practice; (3) collective action—the work that participants do to enact a new practice; and (4) reflexive monitoring—the appraisal work that participants undertake to determine whether the new practice is worth sustaining or how it must be reconfigured to fit their needs [68-70]. All practical applications of the change objectives are linked to the BCTs and NPT domains, as exemplified in Table 2.

**Table 2:** Example matrix for mapping practical applications of performance and change objectives to BCTs^a^ and NPT^b^ domains. The full matrix is available in Multimedia Appendix 1 (Table S2).

Personal determinants and change objectives			Practical application		BCTs as per BCT taxonomy version 1 [64]		NPT domains [68,69]
**PO^c^** **1: accept self-management as a treatment strategy for LBP^d^** **and make the decision to self-manage LBP with support from selfBACK app**							
	Knowledge and awareness: identify positive characteristics of self-management and negative characteristics of provider-dependent behavior and list examples of self-management of LBP Skills: demonstrate the ability to operate selfBACK app Fear avoidance and catastrophizing: recognize fearful thoughts and negative thinking in relation to self-management Self-efficacy: express confidence in the ability to operate selfBACK app Motivation and outcome expectations: express positive feelings or thoughts about engaging in self-management of LBP and expect that self-managing will ease living with LBP and achieving life goals	Introduction session explaining structure and content of app, automatically shown after first log-in and thereafter accessible from Settings Educational messages and quizzes Referral from educational messages to relevant toolbox elements Toolbox elements: resources and interactive tools, knowledge bank, libraries, and pain flare-up relief Visual display of goal accomplishments Rewards for achievements Calendar function Statistics Notifications		5.1 Information about health consequences 5.3 Information about emotional consequences 4.1. Instruction on how to perform the behavior 2.2. Feedback on behavior 10.4. Social reward 7.1. Prompts/cues 15.1. Verbal persuasion about capability		Coherence (gaining an understanding of the condition) Collective action (developing skills) Cognitive participation (engaging with the user to promote uptake) Reflexive monitoring (evaluation and feedback)	
**PO^c^** **14: integrate self-management strategies for LBP^d^** **into daily life**							
	Knowledge and awareness: list ways to integrate self-management of LBP into daily routines Skills: demonstrate the ability to schedule self-management into daily routines Fear avoidance and catastrophizing: recognize fearful and negative thoughts and feelings in relation to integrating self-management into daily routines and recognize own fear avoidance behavior in relation to integrating self-management into daily routines Self-efficacy: express confidence in the ability to integrate self-management of LBP into daily routines Motivation and outcome expectations.: express positive feelings or thoughts about integrating self-management into daily routines and expect that integration of LBP self-management will lead to a healthier, better life	Educational messages and quizzes Referral from educational messages to relevant toolbox elements Toolbox elements: resources and interactive tools, knowledge bank, libraries, and pain flare-up relief Visual display of goal accomplishments Rewards for achievements Calendar function Statistics Notifications Physical activity registration Monitoring of steps Motification system		5.1. Information about health consequences 5.3. Information about emotional consequences 4.1. Instruction on how to perform the behavior 1.5. Review behavioral goals 1.6. Discrepancy between current behavior and goals 10.4. Social reward 2.2. Feedback on behavior 10.5. Social incentive 7.1. Prompts/cues 15.1. Verbal persuasion about capability		Coherence (understanding) Cognitive participation (engaging with the user to promote uptake) Reflexive monitoring (evaluation and feedback)	

^a^BCT: behavior change technique.

^b^NPT: normalization process theory.

^c^PO: performance objective.

^d^LBP: low back pain.

#### Outline of Engagement Strategies

Previous work on DHIs for self-management of LBP identified lack of engagement as a major barrier to use [13,71]. Machado et al [13] advocated the incorporation of strategies to increase engagement by stimulating repeated use, for example, through reminders, gamification, or reward systems. In our systematic review on barriers to and facilitators of engagement in DHIs, we further identified the briefness of information, feedback, tailoring, user-friendliness, design, and layout as facilitators of enhanced engagement in self-management of LBP through DHIs [22]. Gamification, the concept of applying game mechanics to nongame contexts, has been shown to enhance user engagement in DHIs by using many different techniques, for example, badges, progress elements, quizzes, and challenges [72,73]. In addition, gamification in DHIs offers other advantages such as enhancing motivation, making health activities enjoyable and understandable, and improving users’ abilities to self-manage their condition [73]. Therefore, it is thought to contribute to behavior change because of its resemblance with established health BCTs [74]. Our interviews with people with LBP confirmed the empirical findings; a friendly, supportive tone in the app that motivated to do more rather than pointing out insufficiencies was suggested to improve engagement, as well as records of accomplishment and rewards. Feedback on self-management behavior was thought to increase motivation and engagement. Appraisal of the app by the HCP or visibility of a trustworthy source was also important for participants.

**Figure 2:**
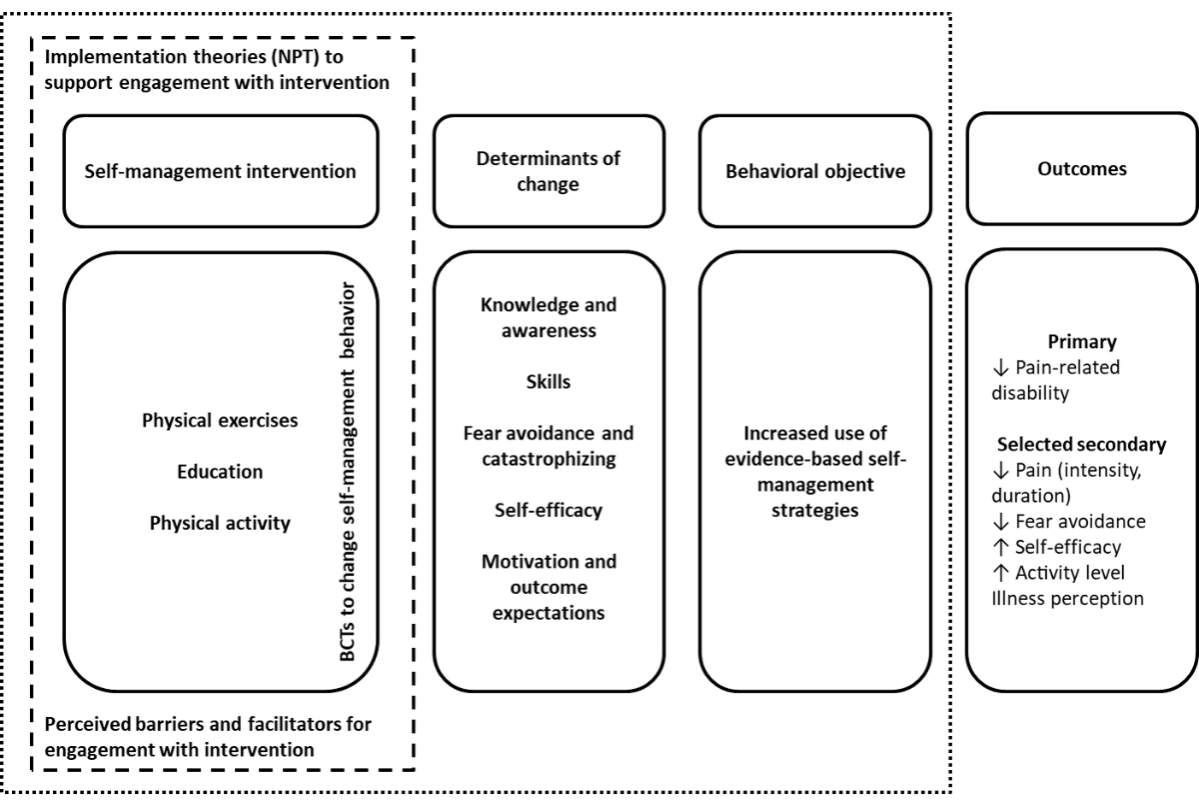
Program logic model of the selfBACK intervention. BCT: behavior change technique; NPT: normalization process theory.

A program logic model bringing together the intended effect outcomes, behavioral outcomes, targeted determinants, and program outputs is presented in Figure 2. This intervention logic model describes the mechanistic pathway from the intervention to the reduction in pain-related disability because of LBP.

### Step 4: Program Production

**Figure 3:**
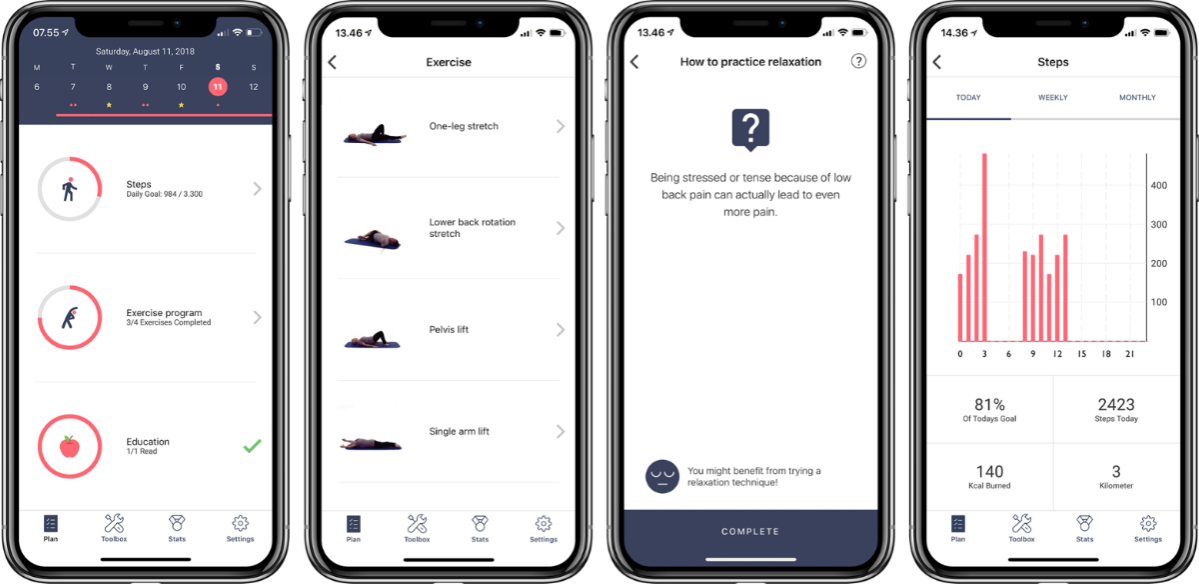
Screenshot of the selfBACK app plan screen showing the 3 main components of a weekly self-management plan and a screenshot of each of the three main components of the self-management intervention: physical exercises, education, and physical activity.

Results from the user journey map tests and continuous user testing of app beta versions provided insights into user needs and potential barriers to use and contributed to improvements in the intervention design and layout (Figure 3). A full description of the app design process and technical specifications, including the personas and user journey map, frontend development, coding, and security measures, are available elsewhere or upon requesting the authors [75].

Results from the feasibility studies improved the activity monitoring from the wristband to the app; established a notification taxonomy taking both achievements and reported barriers into account; and refined the educational content, step count accuracy and goal setting, motivational notifications, and technical barriers to using the app [26]. Finally, the 6-week single-arm pilot study in Denmark and Norway resulted in refinements of recruitment, screening, and app installation procedures as well as effect and process evaluations for the RCT [27].

### Step 5: Implementation Plan

The result of the fifth step was a plan of how we foresaw adoption and implementation in the selfBACK RCT (Table 3). The program use outcomes (adoption and implementation) were formulated with eight performance objectives. Changeable determinants as per NPT [68,69] were identified and crossed with the performance objectives to formulate change objectives. BCTs formed the theoretical foundation for the methods that underpinned the practical strategies for adoption and implementation [64]. A screening and inclusion procedure that lies between the adoption and implementation outcomes has been published [20,27]. On the basis of the results of the pilot study, practical strategies for adoption were refined, including an upscale in the number of recruitment sites or HCPs needed to adopt the intervention to reach the desired number of participants in the RCT.

**Table 3:** Plan for program adoption and implementation of the selfBACK intervention.

Program use outcomes and performance objectives		Determinants for embedment of digital intervention to everyday routine as per NPT^a^ [71,72]	Change objectives	BCTs^b^ to address each change objective as per BCT taxonomy version 1 [67]	Practical strategies
**Adoption use outcome:** **Recruitment sites (HCPs^c^) adopt the selfBACK intervention and participant recruitment procedures**					
	Agree to participate in selfBACK RCT^d^ as recruitment sites	Coherence and cognitive participation	Managers at recruitment sites provide verbal agreement to allow their service to implement selfBACK Managers at recruitment sites allocate resources (time) to support recruitment	1.8. Behavioral contract	Verbal agreement from each recruitment site manager to allow their clinic to implement selfBACK as an add-on to usual care and support participant recruitment to selfBACK RCT^d^ Recruitment site managers to nominate HCPs to receive instruction in recruitment pathway from selfBACK researchers
	Agree to recruit patients to participate in selfBACK RCT	Cognitive participation	HCPs develop an understanding of the purpose, structure, and content of the selfBACK intervention	1.8. Behavioral contract	HCPs receive information about selfBACK RCT and receive information from selfBACK researchers if there are further questions
	HCPs implement recruitment procedures (identification of eligible patients based on RCT inclusion criteria, including practical strategies for establishing contact between patient and selfBACK research team)	Coherence and cognitive participation	HCPs develop an understanding of who eligible patients are HCPs develop skills to initiate recruitment	5.1. Information about health consequences of the intervention 4.1. Instruction on how to perform a behavior	HCPs receive information about selfBACK RCT and receive information from selfBACK researchers if there are further questions HCPs receive recruitment pathway ideas from selfBACK researchers
	HCPs encourage patients to participate in selfBACK RCT as an add-on to usual care	Cognitive participation and collective action	HCPs inform eligible patients about the selfBACK intervention	4.1. Instruction on how to perform a behavior	HCPs give written or verbal information about selfBACK to patients and promote participation verbally
**Implementation use outcome: participants engage in the selfBACK intervention by implementing selfBACK app in daily routines**					
	Participants make sense of the selfBACK intervention	Coherence	Participants develop understanding of the purpose and potential of the selfBACK app	8.1. Behavioral practice/rehearsal	Participants explore the features of the selfBACK app^e^
	Participants build and sustain engagement in the selfBACK intervention	Cognitive participation	Participants initiate regular use of the selfBACK app	8.1. Behavioral practice/rehearsal	Participants launch and use the selfBACK app regularly^e^
	Participants invest efforts and resources in engagement in the selfBACK intervention	Collective action	Participants prioritize regular use of the selfBACK app	8.1. Behavioral practice/rehearsal	Participants launch and use the selfBACK app regularly^e^
	Participants evaluate engagement in the selfBACK intervention	Reflexive monitoring	Participants appraise the selfBACK app and decide to sustain engagement	5.1. Information about health consequences 5.6. Information about emotional consequences	Participants deem selfBACK app as effective or helpful^e^ Participants answer follow-up questionnaires during the trial period

^a^NPT: normalization process theory.

^b^BCT: behavioral change technique.

^c^HCP: health care professional.

^d^RCT: randomized controlled trial.

^e^Practical application is the desired scenario.

## Discussion

### Principal Findings

This study provides a detailed example of using IM to systematically develop a theory and evidence-based app-based intervention for people with nonspecific LBP. There is currently limited literature on the development of complex digital interventions for self-management, and this study should inform future researchers in this evolving field.

During our IM process, we found that the existing knowledge on self-management of LBP is generic with respect to descriptions of intervention content. Clinical practice guidelines for LBP also provide incomplete descriptions of the advice delivered in interventions [76]. In our systematic review, a consistent finding was that a comprehensive description of the development and use of theory was either brief or absent from the included studies [11]. A recent study revealed that there are >700 pain-related smartphone apps available from the main app stores and 61 apps about LBP solely [13]. However, few of these apps have evidence-based content, many have not been rigorously tested for effectiveness, and HCPs and patients are rarely involved in their development [13]. In contrast to the existing apps, selfBACK was developed with a strong theoretical underpinning, involved key stakeholders, integrated feedback from users, and was developed by experts in relevant fields.

As demonstrated in this study, the IM approach details how accessing and using theory can support the development of intervention content as requested in the Medical Research Council’s framework [15] and the CONSORT-EHEALTH checklist [16]. A thorough description of an intervention’s theoretical underpinning increases the understanding of the mechanisms of action and the potential for replication. The application of the IM approach with strong theoretical underpinning will allow for meaningful evaluation of the process of implementing digital self-management of LBP in both people’s daily lives and primary care.

### Strengths and Limitations

A strength of this study is the >2-year development phase following IM. A large and diverse planning group worked continuously on developing, testing, and refining the selfBACK intervention, which made the uniqueness of combining behavior change theory and implementation theory, empirical findings, and state-of-the-art technical solutions such as CBR possible. Rigorous pretesting, an essential part of IM [14], with 2 feasibility studies, a pilot study, and continual user testing involving >200 people, is also a strength of this study. A possible weakness of our study is that we did not fully adhere to the IM protocol. Our needs assessment and the resulting logic model of the problem encountered self-management of LBP exclusively from the target population’s perspective. The IM protocol advocates a multilevel approach, taking environmental factors and the potential influence of environmental agents into account [14].

### Conclusions

This paper reports a detailed example of the application of IM in the development of a theory-driven complex DHI designed to support self-management for people with LBP. Although IM is a time-intensive collaborative process, this report of the range of methods used provides a transparent account of the development process and a blueprint for designing and creating future DHIs for self-management.

## Appendix

Multimedia Appendix 1 Matrix of change objectives for the behavioral outcome “To increase use of evidence-based self-management strategies” and Mapping practical applications of performance and change objectives to behavior change techniques and normalization process theory domains.

## Abbreviations

BCT: behavior change technique
CBR: case-based reasoning
CONSORT-EHEALTH: Consolidated Standards of Reporting Trials of Electronic and Mobile Health Applications and Online Telehealth
DHI: digital health intervention
HCP: health care professional
IM: intervention mapping
LBP: low back pain
NPT: normalization process theory
RCT: randomized controlled trial
SMP: self-management plan
TDF: Transtheoretical Domains Framework
