# Supplementary material for: Using Intervention Mapping to Develop a Decision Support System–Based Smartphone App (selfBACK) to Support Self-management of Nonspecific Low Back Pain: Development and Usability Study
Source: J Med Internet Res. 2022 Jan 24;24(1):e26555. doi: 10.2196/26555 (PMC8822424; doi:10.2196/26555)
Supplement: Multimedia Appendix 1 [file jmir_v24i1e26555_app1.docx]

Table S1: Matrix of change objectives for the behavioural outcome ‘To increase use of evidence-based self-management strategies’.

|  | Personal determinants | | | | |
| --- | --- | --- | --- | --- | --- |
|  | Knowledge and awareness^a^ | Skills^b^ | Fear-avoidance and catastrophizing^c^ | Self-efficacy^d^ | Motivation and outcome expectations^e^ |
| **PO^f^1: Accept self-management as treatment strategy for LBP and make the decision to self-manage LBP with support from selfBACK app** | | | | | |
|  | **Change objectives** | | | | |
|  | - Identify positive characteristics of self-management and negative characteristics of provider dependent behaviour - List examples of self-management of LBP | - Demonstrate ability to operate selfBACK app | - Recognise fearful thoughts and negative thinking in relation to self-management | - Express confidence in ability to operate selfBACK app | - Express positive feelings or thoughts about engaging in self-management of LBP - Expect that self-managing will ease living with LBP and achieving life goals |
| **PO^f^2: Make the decision to be physically active** | | | | | |
|  | **Change objectives** | | | | |
|  | - Explain positive effects of physical activity and negative effects of sedentary behaviour |  | - Recognise fearful thoughts and negative thinking in relation to physical activity - Recognise own fear-avoidance behaviour in relation to physical activity | - Express confidence in ability to be physically active | - Express positive feelings or thoughts about physical activity - Expect that physical activity will ease living with LBP |
| **PO^f^3: Make the decision to perform physical exercises for LBP** | | | | | |
|  | **Change objectives** | | | | |
|  | - Identify positive effects of physical exercises for LBP |  | - Recognise fearful thoughts and negative feelings in relation to physical exercises for LBP - Recognise own fear-avoidance behaviour in relation to physical exercises for LBP | - Express confidence in ability to be perform physical exercises for LBP | - Express positive feelings or thoughts about physical exercises for LBP - Expect that physical exercises for LBP will ease living with LBP |
| **PO^f^4: Make the decision to use pain coping strategies for LBP** | | | | | |
|  | **Change objectives** | | | | |
|  | - Identify positive effects of pain coping strategies for LBP |  | - State that pain coping strategies will help to overcome fearful thoughts and negative thinking | - Express confidence in ability to utilise pain coping strategies | - Express positive feelings or thoughts about pain coping strategies for LBP - Expect that pain coping strategies will ease living with LBP |
| **PO^f^5: Make the decision to use good sleep hygiene strategies** | | | | | |
|  | **Change objectives** | | | | |
|  | - Describe positive effects of good sleep hygiene |  | - State that sleep will help to cope with LBP and negative thoughts | - Express confidence in ability to utilise good sleep hygiene strategies | - Express positive feelings or thoughts about good sleep hygiene strategies - Expect that a good sleep hygiene will ease living with LBP |
| **PO^f^6: Select physical activities** | | | | | |
|  | **Change objectives** | | | | |
|  | - Identify different types of physical activity and relative advantages/disadvantages of each type in relation to LBP - State personal preferences for physical activities |  |  | - Express confidence in ability to select physical activities |  |
| **PO^f^7: Select physical exercises for LBP** | | | | | |
|  | **Change objectives** | | | | |
|  | - List examples of physical exercises for LBP - Give examples of modifications or increased/ decreased difficulty of exercises - State personal favourite physical exercises for LBP |  |  | - Express confidence in ability to select physical exercises for LBP |  |
| **PO^f^8: Select pain coping strategies for LBP** | | | | | |
|  | **Change objectives** | | | | |
|  | - List examples of pain coping strategies for LBP - Identify personal preference according to relevance |  |  | - Express confidence in ability to select pain coping strategies for LBP |  |
| **PO^f^9: Perform physical activities** | | | | | |
|  | **Change objectives** | | | | |
|  | - List where and when physical activities can be performed - Describe duration, intensity and frequency of physical activities | - Demonstrate ability to perform physical activities | - Acknowledge that performing physical activities might result in temporary increase in LBP | - Express confidence in ability to perform physical activities | - Express positive feelings or thoughts about performing physical activities - Expect that performing physical activities will lead to healthier, better life |
| **PO^f^10: Perform physical exercises for LBP** | | | | | |
|  | **Change objectives** | | | | |
|  | - List where and when physical exercises for LBP can be performed - Describe duration, intensity and frequency of physical exercises for LBP | - Demonstrate ability to perform physical exercises for LBP | - Acknowledge that performing physical exercises might result in temporary increase in LBP | - Express confidence in ability to perform physical exercises for LBP | - Express positive feelings or thoughts about performing physical exercises for LBP - Expect that performing physical exercises for LBP will lead to healthier, better life |
| **PO^f^11: Use pain coping strategies for LBP** | | | | | |
|  | **Change objectives** | | | | |
|  | - Describe how and when to use pain coping strategies for LBP | - Demonstrate ability to use pain coping strategies for LBP | - Acknowledge that utilising some pain coping strategies might result in temporary increase in LBP | - Express confidence in ability to utilise pain coping strategies for LBP | - Express positive feelings or thoughts about utilising pain coping strategies for LBP - Expect that utilising pain coping strategies will ease living with LBP |
| **PO^f^12: Use goal setting and pacing** | | | | | |
|  | **Change objectives** | | | | |
|  | - Describe how and when to use goal setting for LBP self-management | - Demonstrate ability to use goal setting and pacing techniques | - Acknowledge that goal setting and pacing is helpful for LBP self-management | - Express confidence in ability to utilise goal setting and pacing for LBP self-management | - Express positive feelings or thoughts about utilising goal setting and pacing for LBP self-management - Expect that utilising goal setting and pacing will ease living with LBP |
| **PO^f^13: Use good sleep hygiene strategies** | | | | | |
|  | **Change objectives** | | | | |
|  | - List examples of good sleep hygiene - State personal strategy for good sleep hygiene | - Demonstrate ability to use sleep hygiene strategies |  | - Express confidence in ability to utilise good sleep hygiene strategies | - Express positive feelings or thoughts about utilising good sleep hygiene strategies - Expect that having a good sleep hygiene will ease living with LBP |
| **PO^f^14: Integrate self-management strategies for LBP into daily life** | | | | | |
|  | **Change objectives** | | | | |
|  | - List ways to integrate self-management of LBP into daily routines | - Demonstrate ability to schedule self-management into daily routines | - Recognise fearful and negative thoughts and feelings in relation to integrating self-management into daily routines - Recognise own fear-avoidance behaviour in relation integrating self-management into daily routines | - Express confidence in ability to integrate self-management of LBP into daily routines | - Express positive feelings or thoughts about integrating self-management into daily routines - Expect that integration of LBP self-management will lead to healthier, better life |

^a^Increase knowledge of self-management behaviour
^b^Develop ability to engage in self-management behaviour ^c^Reduce fear or negative expectancies about engaging in self-management behaviour ^d^Improve perceived ability to uptake and engage in self-management behaviour ^e^Improve autonomous motivation to engage in self-management behaviour and improve expectations to the outcome of self-management behaviour
^f^PO: Performance objective

Table S2: Mapping practical applications of performance and change objectives to BCTs^a^ and NPT^b^ domains

| Personal determinants and change objectives | Practical application | BCTs as per BCT Taxonomy v1[64] | NPT domains[68, 69] |
| --- | --- | --- | --- |
| **PO^c^1: Accept self-management as treatment strategy for LBP and make the decision to self-manage LBP with support from selfBACK app** | | | |
| KA^d^.1a Identify positive characteristics of self-management and negative characteristics of provider dependent behaviour  KA^d^.1b List examples of self-management of LBP  S^e^.1 Demonstrate ability to operate selfBACK app  FAC^f^.1 Recognise fearful thoughts and negative thinking in relation to self-management  SE^g^.1 Express confidence in ability to operate selfBACK app  MOE^h^.1a Express positive feelings or thoughts about engaging in self-management of LBP  MOE^h^.1b Expect that self-managing will ease living with LBP and achieving life goals | Introduction session explaining structure and content of app, automatically shown after first login and thereafter accessible from ‘Settings’ | 5.1 Information about health consequences | Coherence (gaining an understanding of the condition) |
|  | Educational messages and quizzes | 5.1 Information about health consequences  5.3 Information about emotional consequences | Coherence (gaining an understanding of the condition) |
|  | Referral from educational messages to relevant toolbox elements | 5.1 Information about health consequences  5.3 Information about emotional consequences | Coherence (gaining an understanding of the condition) |
|  | Toolbox elements: resources and interactive tools, knowledge bank, libraries, pain flare-up relief | 4.1 Instruction on how to perform the behaviour  5.1 Information about health consequences  5.3 Information about emotional consequences | Collective Action (developing skills) |
|  | Visual display of goal accomplishments | 2.2. Feedback on behaviour | Cognitive Participation (engaging with the user to promote uptake) |
|  | Rewards for achievements | 2.2 Feedback on behaviour  10.4 Social reward | Reflexive Monitoring (evaluation and feedback) |
|  | Calendar function | 7.1 Prompts/cues | Cognitive Participation (engaging with the user to promote uptake) |
|  | Statistics | 2.2. Feedback on behaviour | Reflexive Monitoring (evaluation and feedback) |
|  | Notifications | 2.2. Feedback on behaviour  7.1 Prompts/cues  15.1 Verbal persuasion about capability | Cognitive Participation (engaging with the user to promote uptake) |
| **PO^c^2: Make the decision to be physically active** | | | |
| KA^d^.2 Explain positive effects of physical activity and negative effects of sedentary behaviour  FAC^f^.2a Recognise fearful thoughts and negative thinking in relation to physical activity  FAC^f^.2b Recognise own fear-avoidance behaviour in relation to physical activity  SE^g^.2 Express confidence in ability to be physically active  MOE^h^.2a Express positive feelings or thoughts about physical activity  MOE^h^.2b Expect that physical activity will ease living with LBP | Educational messages and quizzes | 5.1 Information about health consequences  5.3 Information about emotional consequences | Coherence (gaining an understanding of the condition and treatments) |
|  | Referral from educational messages to relevant toolbox elements | 5.1 Information about health consequences  5.3 Information about emotional consequences | Coherence (gaining an understanding of the condition and treatments) |
|  | Toolbox element: knowledge bank | 4.1 Instruction on how to perform the behaviour  5.1 Information about health consequences  5.3 Information about emotional consequences | Coherence (gaining an understanding of the condition and treatments) |
|  | Visual display of goal accomplishments | 1.7 Review outcome goal(s)  1.6 Discrepancy between current behaviour and goals | Cognitive Participation (engaging with the user to promote uptake) |
|  | Rewards for achievements | 10.4 Social reward | Reflexive Monitoring (evaluation and feedback) |
|  | Statistics | 1.5 Review behavioural goals  1.6 Discrepancy between current behaviour and goal | Reflexive Monitoring (evaluation and feedback) |
|  | Notifications | 2.2. Feedback on behaviour  7.1 Prompts/cues  15.1 Verbal persuasion about capability | Cognitive Participation (engaging with the user to promote uptake) |
|  | Physical activity registration | 2.2 Feedback on behaviour | Reflexive Monitoring (evaluation and feedback) |
|  | Monitoring of steps | 7.1 Prompts/cues  2.2 Feedback on behaviour | Reflexive Monitoring (evaluation and feedback) |
|  | “Motification” system | 15.1 Verbal persuasion about capability  7.1 Prompts/cues | Cognitive Participation (engaging with the user to promote uptake) |
| **PO^c^3: Make the decision to perform physical exercises for LBP** | | | |
| KA^d^.3 Identify positive effects of physical exercises for LBP  FAC^f^.3a Recognise fearful thoughts and negative feelings in relation to physical exercises for LBP  FAC^f^.3b Recognise own fear-avoidance behaviour in relation to physical exercises for LBP  SE^g^.3 Express confidence in ability to be perform physical exercises for LBP  MOE^h^.3a Express positive feelings or thoughts about physical exercises for LBP  MOE^h^.3b Expect that physical exercises for LBP will ease living with LBP | Educational messages and quizzes | 5.1 Information about health consequences  5.3 Information about emotional consequences | Coherence (gaining an understanding of the condition and treatments) |
|  | Referral from educational messages to relevant toolbox elements | 5.1 Information about health consequences  5.3 Information about emotional consequences | Coherence (gaining an understanding of the condition and treatments) |
|  | Toolbox element: knowledge bank | 4.1 Instruction on how to perform the behaviour  5.1 Information about health consequences  5.3 Information about emotional consequences | Coherence (gaining an understanding of the condition and treatments) |
|  | Visual display of goal accomplishments | 1.7 Review outcome goal(s)  1.6 Discrepancy between current behaviour and goal | Cognitive Participation (engaging with the user to promote uptake) |
|  | Rewards for achievements | 10.4 Social reward | Reflexive Monitoring (evaluation and feedback) |
|  | Statistics | 1.5 Review behavioural goals  1.6 Discrepancy between current behaviour and goals | Reflexive Monitoring (evaluation and feedback) |
|  | Notifications | 7.1 Prompts/cues  2.2. Feedback on behaviour  15.1 Verbal persuasion about capability | Cognitive Participation (engaging with the user to promote uptake) |
| **PO^c^4: Make the decision to use pain coping strategies for LBP** | | | |
| KA^d^.4 Identify positive effects of pain coping strategies for LBP  FAC^f^.4 State that pain coping strategies will help to overcome fearful thoughts and negative thinking  SE^g^.4 Express confidence in ability to utilise pain coping strategies  MOE^h^.4a Express positive feelings or thoughts about pain coping strategies for LBP  MOE^h^.4b Expect that pain coping strategies will ease living with LBP | Educational messages and quizzes | 5.1 Information about health consequences  5.3 Information about emotional consequences | Coherence (gaining an understanding of the condition and treatments) |
|  | Referral from educational messages to relevant toolbox elements | 5.1 Information about health consequences  5.3 Information about emotional consequences | Coherence (gaining an understanding of the condition and treatments) |
|  | Toolbox element: knowledge bank | 4.1 Instruction on how to perform the behaviour  5.1 Information about health consequences  5.3 Information about emotional consequences | Coherence (gaining an understanding of the condition and treatments) |
|  | Visual display of goal accomplishments | 1.5 Review behavioural goals  1.6 Discrepancy between current behaviour and goal | Cognitive Participation (engaging with the user to promote uptake) |
|  | Rewards for achievements | 10.4 Social reward | Reflexive Monitoring (evaluation and feedback) |
|  | Statistics | 10.5 Social incentive  1.5 Review behavioural goals  1.6 Discrepancy between current behaviour and goals | Reflexive Monitoring (evaluation and feedback) |
|  | Notifications | 7.1 Prompts/cues  2.2 Feedback on behaviour  15.1 Verbal persuasion about capability | Cognitive Participation (engaging with the user to promote uptake) |
| **PO^c^5: Make the decision to use good sleep hygiene strategies** | | | |
| KA^d^.5 Describe positive effects of good sleep hygiene  FAC^f^.5 State that sleep will help to cope with LBP and negative thoughts  SE^g^.5 Express confidence in ability to utilise good sleep hygiene strategies  MOE^h^.5a Express positive feelings or thoughts about good sleep hygiene strategies  MOE^h^.5b Expect that a good sleep hygiene will ease living with LBP | Educational messages and quizzes | 5.1 Information about health consequences  5.3 Information about emotional consequences | Coherence (gaining an understanding of the condition and treatments) |
|  | Referral from educational messages to relevant toolbox elements | 5.1 Information about health consequences  5.3 Information about emotional consequences | Coherence (gaining an understanding of the condition and treatments) |
|  | Toolbox element: knowledge bank | 4.1 Instruction on how to perform the behaviour  5.1 Information about health consequences  5.3 Information about emotional consequences | Coherence (gaining an understanding of the condition and treatments) |
| **PO^c^6: Select physical activities** | | | |
| KA^d^.6a Identify different types of physical activity and relative advantages/disadvantages of each type in relation to LBP  KA^d^.6b State personal preferences for physical activities  SE^g^.6 Express confidence in ability to select physical activities | Tailoring session | 1.1 Goal setting (behaviour)  1.8 behavioural contract | Coherence (setting goals that are deemed suitable for the individual) |
|  | Educational messages and quizzes | 5.1 Information about health consequences  5.3 Information about emotional consequences | Coherence (gaining an understanding of the condition and treatments) |
|  | Referral from educational messages to relevant toolbox elements | 5.1 Information about health consequences  5.3 Information about emotional consequences | Coherence (gaining an understanding of the condition and treatments) |
|  | Toolbox elements: knowledge bank, library of messages and quizzes | 4.1 Instruction on how to perform the behaviour  5.1 Information about health consequences  5.3 Information about emotional consequences | Coherence (gaining an understanding of the condition and treatments) |
| **PO^c^7: Select physical exercises for LBP** | | | |
| KA^d^.7a List examples of physical exercises for LBP  KA^d^.7b Give examples of modifications or increased/ decreased difficulty of exercises  KA^d^.7c State personal favourite physical exercises for LBP  SE^g^.7 Express confidence in ability to select physical exercises for LBP | Tailoring session | 5.1 Information about health consequences  5.3 Information about emotional consequences  1.8 Behavioural contract | Coherence (setting goals that are deemed suitable for the individual) |
|  | Educational messages and quizzes | 5.1 Information about health consequences  5.3 Information about emotional consequences | Coherence (gaining an understanding of the condition and treatments) |
|  | Referral from educational messages to relevant toolbox elements | 6.1 Demonstration of the behaviour  5.1 Information about health consequences  5.3 Information about emotional consequences | Coherence (gaining an understanding of the condition and treatments) |
|  | Toolbox elements: knowledge bank, library of physical exercises | 4.1 Instruction on how to perform the behaviour  5.1 Information about health consequences  5.3 Information about emotional consequences | Coherence (gaining an understanding of the condition and treatments) |
| **PO^c^8: Select pain coping strategies for LBP** | | | |
| KA^d^.8a List examples of pain coping strategies for LBP  KA^d^.8b Identify personal preference according to relevance  SE^g^.8 Express confidence in ability to select pain coping strategies for LBP | Tailoring session | 1.8 Behavioural contract | Coherence (setting goals that are deemed suitable for the individual) |
|  | Educational messages and quizzes | 5.1 Information about health consequences  5.3 Information about emotional consequences | Coherence (gaining an understanding of the condition and treatments) |
|  | Referral from educational messages to relevant toolbox elements | 5.1 Information about health consequences  5.3 Information about emotional consequences | Coherence (gaining an understanding of the condition and treatments) |
|  | Toolbox elements: knowledge bank, libraries, resources and interactive tools, pain flare-up relief | 4.1 Instruction on how to perform the behaviour  5.1 Information about health consequences  5.3 Information about emotional consequences | Coherence (gaining an understanding of the condition and treatments) |
| **PO^c^9: Perform physical activities** | | | |
| KA^d^.9a List where and when physical activities can be performed  KA^d^.9b Describe duration, intensity and frequency of physical activities  S^e^.9 Demonstrate ability to perform physical activities  FAC^f^.9 Acknowledge that performing physical activities might result in temporary increase in LBP  SE^g^.9 Express confidence in ability to perform physical activities  MOE^h^.9a Express positive feelings or thoughts about performing physical activities  MOE^h^.9b Expect that performing physical activities will lead to healthier, better life | Educational messages and quizzes | 5.1 Information about health consequences  5.3 Information about emotional consequences | Coherence (gaining an understanding of the condition and treatments) |
|  | Referral from educational messages to relevant toolbox elements | 5.1 Information about health consequences  5.3 Information about emotional consequences | Coherence (gaining an understanding of the condition and treatments) |
|  | Toolbox elements: knowledge bank, resources and interactive tools | 4.1 Instruction on how to perform the behaviour  5.1 Information about health consequences  5.3 Information about emotional consequences | Coherence (gaining an understanding of the condition and treatments) |
|  | Visual display of goal accomplishments | 1.5 Review behavioural goals  1.6 Discrepancy between current behaviour and goal | Cognitive Participation (engaging with the user to promote uptake) |
|  | Rewards for achievements | 10.4 Social reward  1.6 Discrepancy between current behaviour and goal | Reflexive Monitoring (evaluation and feedback) |
|  | Calendar function | 2.2 Feedback on behaviour | Cognitive Participation (engaging with the user to promote uptake) |
|  | Statistics | 10.5 Social incentive  1.5 Review behavioural goals  1.6 Discrepancy between current behaviour and goals | Reflexive Monitoring (evaluation and feedback) |
|  | Notifications | 7.1 Prompts/cues  2.2. Feedback on behaviour | Cognitive Participation (engaging with the user to promote uptake) |
|  | Physical activity registration | 2.2 Feedback on behaviour | Cognitive Participation (engaging with the user to promote uptake) |
|  | Monitoring of steps | 1.5 Review behaviour goals  1.6 Discrepancy between current behaviour and goals  2.2. Feedback on behaviour | Reflexive Monitoring (evaluation and feedback) |
|  | “Motification” system | 15.1 Verbal persuasion about capability  7.1 Prompts/cues | Cognitive Participation (engaging with the user to promote uptake) |
| **PO^c^10: Perform physical exercises for LBP** | | | |
| KA^d^.10a List where and when physical exercises for LBP can be performed  KA^d^.10b Describe duration, intensity and frequency of physical exercises for LBP  S^e^.10 Demonstrate ability to perform physical exercises for LBP  FAC^f^.10 Acknowledge that performing physical exercises might result in temporary increase in LBP  SE^g^.10 Express confidence in ability to perform physical exercises for LBP  MOE^h^.10a Express positive feelings or thoughts about performing physical exercises for LBP  MOE^h^10.b Expect that performing physical exercises for LBP will lead to healthier, better life | Tailoring session | 8.7 Graded tasks | Coherence (setting goals that are deemed suitable for the individual) |
|  | Educational messages and quizzes | 5.1 Information about health consequences  5.3 Information about emotional consequences | Coherence (gaining an understanding of the condition and treatments) |
|  | Referral from educational messages to relevant toolbox elements | 5.1 Information about health consequences  5.3 Information about emotional consequences | Coherence (gaining an understanding of the condition and treatments) |
|  | Toolbox elements: knowledge bank, library of physical exercises, resources and interactive tools, pain flare-up relief | 4.1 Instruction on how to perform the behaviour  5.1 Information about health consequences  5.3 Information about emotional consequences | Coherence (gaining an understanding of the condition and treatments) |
|  | Physical exercise instruction through videos, text and number of sets and repetitions | 6.1 Demonstration of behaviour | Collective Action (developing skills) |
|  | Swap function for physical exercises including providing reason for wanting a swap | 8.7 Graded tasks | Collective Action (interactional workability) |
|  | Visual display of goal accomplishments | 1.5 Review behavioural goals  1.6 Discrepancy between current behaviour and goals | Cognitive Participation (engaging with the user to promote uptake) |
|  | Rewards for achievements | 10.4 Social reward  1.6 Discrepancy between current behaviour and goal | Reflexive Monitoring (evaluation and feedback) |
|  | Calendar function | 2.2 Feedback on behaviour | Cognitive Participation (engaging with the user to promote uptake) |
|  | Statistics | 10.5 Social incentive  1.5 Review behavioural goals  1.6 Discrepancy between current behaviour and goals | Reflexive Monitoring (evaluation and feedback) |
|  | Notifications | 7.1 Prompts/cues  2.2. Feedback on behaviour | Cognitive Participation (engaging with the user to promote uptake) |
| **PO^c^11: Use pain coping strategies for LBP** | | | |
| KA^d^.11 Describe how and when to use pain coping strategies for LBP  S^e^.11 Demonstrate ability to use pain coping strategies for LBP  FAC^f^.11 Acknowledge that utilising some pain coping strategies might result in temporary increase in LBP  SE^g^.11 Express confidence in ability to utilise pain coping strategies for LBP  MOE^h^.11a Express positive feelings or thoughts about utilising pain coping strategies for LBP  MOE^h^.11b Expect that utilising pain coping strategies will ease living with LBP | Tailoring session | 4.1 Instruction on how to perform the behaviour | Coherence (setting goals that are deemed suitable for the individual) |
|  | Educational messages and quizzes  Referral from educational messages to relevant toolbox elements | 5.1 Information about health consequences  5.3 Information about emotional consequences | Coherence (gaining an understanding of the condition and treatments) |
|  | Toolbox elements: knowledge bank, resources and interactive tools; libraries | 4.1 Instruction on how to perform the behaviour  5.1 Information about health consequences  5.3 Information about emotional consequences | Coherence (gaining an understanding of the condition and treatments) |
|  | Visual display of goal accomplishments | 1.5 Review behavioural goals  1.6 Discrepancy between current behaviour and goals | Cognitive Participation (engaging with the user to promote uptake) |
|  | Rewards for achievements | 10.4 Social reward  1.6 Discrepancy between current behaviour and goal | Reflexive Monitoring (evaluation and feedback) |
|  | Calendar function | 2.2 Feedback on behaviour | Cognitive Participation (engaging with the user to promote uptake) |
|  | Statistics | 10.5 Social incentive  1.5 Review behavioural goals  1.6 Discrepancy between current behaviour and goals | Reflexive Monitoring (evaluation and feedback) |
|  | Notifications | 7.1 Prompts/cues  2.2. Feedback on behaviour | Cognitive Participation (engaging with the user to promote uptake) |
|  | Physical activity registration | 2.2 Feedback on behaviour | Cognitive Participation (engaging with the user to promote uptake) |
|  | Monitoring of steps | 1.5 Review behaviour goals  1.6 Discrepancy between current behaviour and goals  2.2. Feedback on behaviour | Reflexive Monitoring (evaluation and feedback) |
|  | “Motification” system | 15.1 Verbal persuasion about capability  7.1 Prompts/cues | Cognitive Participation (engaging with the user to promote uptake) |
|  | Tailoring session | 1.1 Goal setting  1.4 Action planning | Coherence (setting goals that are deemed suitable for the individual) |
| **PO^c^12: Use goal setting and pacing** | | | |
| KA^d^.12 Describe how and when to use goal setting for LBP self-management  S^e^.12 Demonstrate ability to use goal setting and pacing techniques  FAC^f^.12 Acknowledge that goal setting and pacing is helpful for LBP self-management  SE^g^.12 Express confidence in ability to utilise goal setting and pacing for LBP self-management  MOE^h^.12a Express positive feelings or thoughts about utilising goal setting and pacing for LBP self-management  MOE^h^.12b Expect that utilising goal setting and pacing will ease living with LBP | Educational messages and quizzes | 5.1 Information about health consequences  5.3 Information about emotional consequences | Coherence (gaining an understanding of the condition and treatments) |
|  | Referral from educational messages to relevant toolbox elements | 5.1 Information about health consequences  5.3 Information about emotional consequences | Coherence (gaining an understanding of the condition and treatments) |
|  | Toolbox elements: knowledge bank, resources and interactive tools | 4.1 Instruction on how to perform the behaviour  5.1 Information about health consequences  5.3 Information about emotional consequences | Coherence (gaining an understanding of the condition and treatments) |
|  | Visual display of goal accomplishments | 1.5 Review behavioural goals  1.6 Discrepancy between current behaviour and goals | Cognitive Participation (engaging with the user to promote uptake) |
|  | Rewards for achievements | 10.4 Social reward  1.6 Discrepancy between current behaviour and goal | Reflexive Monitoring (evaluation and feedback) |
|  | Calendar function | 2.2 Feedback on behaviour | Cognitive Participation (engaging with the user to promote uptake) |
|  | Statistics | 10.5 Social incentive  1.5 Review behavioural goals  1.6 Discrepancy between current behaviour and goals | Reflexive Monitoring (evaluation and feedback) |
|  | Notifications | 7.1 Prompts/cues  2.2. Feedback on behaviour | Cognitive Participation (engaging with the user to promote uptake) |
|  | Physical activity registration | 2.2 Feedback on behaviour | Cognitive Participation (engaging with the user to promote uptake) |
|  | Monitoring of steps | 1.5 Review behavioural goals  1.6 Discrepancy between current behaviour and goal  2.2. Feedback on behaviour | Reflexive Monitoring (evaluation and feedback) |
|  | “Motification” system | 15.1 Verbal persuasion about capability  7.1 Prompts/cues | Cognitive Participation (engaging with the user to promote uptake) |
|  | Educational messages and quizzes | 5.1 Information about health consequences  5.3 Information about emotional consequences | Coherence (developing understanding) |
| **PO^c^13: Use good sleep hygiene strategies** | | | |
| KA^d^.13a List examples of good sleep hygiene  KA^d^.13b State personal strategy for good sleep hygiene  S^e^.13 Demonstrate ability to use sleep hygiene strategies  SE^g^.13 Express confidence in ability to utilise good sleep hygiene strategies  MOE^h^.13a Express positive feelings or thoughts about utilising good sleep hygiene strategies  MOE^h^.13b Expect that having a good sleep hygiene will ease living with LBP | Referral from educational messages to bedtime reminder tool and sleep hygiene tips | 5.1 Information about health consequences  5.3 Information about emotional consequences | Coherence (developing understanding) |
|  | Toolbox elements: pain relief flare-up, resources and interactive tools, knowledge bank | 4.1 Instruction on how to perform the behaviour  5.1 Information about health consequences  5.3 Information about emotional consequences | Coherence (developing understanding) |
|  | Tailoring session | 4.1 Instruction on how to perform the behaviour  11.2 Resolve negative emotions | Coherence (setting goals that are deemed suitable for the individual) |
| **PO^c^14: Integrate self-management strategies for LBP into daily life** | | | |
| KA^d^.14 List ways to integrate self-management of LBP into daily routines  S^e^.14 Demonstrate ability to schedule self-management into daily routines  FAC^f^.14a Recognise fearful and negative thoughts and feelings in relation to integrating self-management into daily routines  FAC^f^.14b Recognise own fear-avoidance behaviour in relation integrating self-management into daily routines  SE^g^.14 Express confidence in ability to integrate self-management of LBP into daily routines  MOE^h^.14a Express positive feelings or thoughts about integrating self-management into daily routines  MOE^h^.14b Expect that integration of LBP self-management will lead to healthier, better life | Educational messages and quizzes | 5.1 Information about health consequences  5.3 Information about emotional consequences | Coherence (understanding) |
|  | Referral from educational messages to relevant toolbox elements | 5.1 Information about health consequences  5.3 Information about emotional consequences | Coherence (understanding) |
|  | Toolbox elements: resources and interactive tools, knowledge bank, libraries, pain flare-up relief | 4.1 Instruction on how to perform the behaviour  5.1 Information about health consequences  5.3 Information about emotional consequences | Coherence (understanding) |
|  | Visual display of goal accomplishments | 1.5 Review behavioural goals  1.6 Discrepancy between current behaviour and goals | Cognitive Participation (engaging with the user to promote uptake) |
|  | Rewards for achievements | 10.4 Social reward  1.6 Discrepancy between current behaviour and goal | Reflexive Monitoring (evaluation and feedback) |
|  | Calendar function | 2.2 Feedback on behaviour | Cognitive Participation (engaging with the user to promote uptake) |
|  | Statistics | 10.5 Social incentive  1.5 Review behavioural goals  1.6 Discrepancy between current behaviour and goals | Reflexive Monitoring (evaluation and feedback) |
|  | Notifications | 7.1 Prompts/cues  2.2. Feedback on behaviour | Cognitive Participation (engaging with the user to promote uptake) |
|  | Physical activity registration | 2.2 Feedback on behaviour | Cognitive Participation (engaging with the user to promote uptake) |
|  | Monitoring of steps | 1.5 Review behavioural goals  1.6 Discrepancy between current behaviour and goal  2.2. Feedback on behaviour | Reflexive Monitoring (evaluation and feedback) |
|  | “Motification” system | 15.1 Verbal persuasion about capability  7.1 Prompts/cues | Cognitive Participation (engaging with the user to promote uptake) |

^a^BCT: behavior change technique
^b^NPT: Normalization Process Theory ^c^PO: performance objective
^d^KA: knowledge and awareness
^e^S: skills
^f^FAC: fear avoidance and catastrophizing
^g^SE: self-efficacy
^h^MOE: motivation and outcome expectations
